# The effectiveness of serious games for training of attention and executive functions: a systematic review

**DOI:** 10.3389/frcha.2026.1801077

**Published:** 2026-05-11

**Authors:** Vincenza Tommasi, Antonella Spinelli, Valentina Siena, Chiara Romano, Francesca Bruno, Giuliana Nardacchione, Chiara Valeria Marinelli

**Affiliations:** Cognitive and Affective Neuroscience Lab (CANLab), University of Foggia, Foggia, Italy

**Keywords:** ADHD, attention, autism, behavioral disorders, executive functions, neuropsychological skills, serious games

## Abstract

**Introduction:**

Evidence suggests that Serious games (SG) can be effective for improving specific neuropsychological skills. This systematic review examines the effectiveness of SGs for training attention and executive functions (EFs) in children and the degree of “far transfer” from digital training to real-world academic and behavioral outcomes.

**Methods:**

The systematic review was conducted according to the PRISMA (2020) guidelines on Scopus, PubMed, EBSCO, and Web of Science databases. Only studies on school-aged population with a pre- and post-training assessment of attention and EFs and with a control group were included.

**Results:**

The screening process yielded nine relevant studies. SGs improve attention and EFs, such as working memory, cognitive flexibility and inhibition in typically developing children, as well as in children with ADHD, intellectual disabilities, behavioral disorders, autism and difficulty with inhibitory control. SGs demonstrate promising improvements primarily in near-transfer.

**Discussion:**

Although the immediate benefits are evident in many cases, the transfer to untrained skills (far transfer) and the long-term effects remain limited and require further investigation. Motivation and engagement were often qualitatively inferred rather than directly measured. The effectiveness data were discussed in relation to the type of neurodevelopmental deficit.

## Highlights

• Effective serious games for typical and atypical children.

• Potential of serious game for strengthening attention and executive functions.

## 1 Introduction

In the contemporary digital world, children are exposed to technology from an ever-earlier age. This is a precious opportunity to pivot technological exposure toward rehabilitative aims, transforming digital devices into a vehicle for cognitive enhancement. Traditional perspectives perceive learning as a rigorous obligation and play as mere recreation. Instead, play constitutes a natural form of learning, and effective learning environments often mirror the interactive dynamics of play (1). In this sense, the challenging nature of digital gaming can be defined as “hard fun” (2), and the effort in gaming becomes educational/rehabilitative.

There are several type of digital games with various therapeutic formats tailored to specific clinical needs, such as: 1) exergames, which integrate physical activity and movement into the gaming experience to foster both motor and cognitive health (67); 2) cognitive training software specifically designed to preserve or enhance cognitive functions, such as memory, learning, and attention (68); 3) computerized cognitive behavioral therapy (CBT) games, which adapt evidence-based psychological protocols, such as exposure therapy, into an engaging digital format (69); and 4) immersive technologies, such as Virtual Reality (VR) and Augmented Reality (AR), which provide high-fidelity environments for the assessment and training of executive control (70, 71). These devices have dynamically adapted to technological progress (72), evolving from basic digital interfaces to sophisticated 3D simulations (73).

Serious Games (SGs) are digital games designed for educational and/or therapeutic purposes, such as training, rehabilitation and skills improvement (74, 75). Based on gamification principles, SGs integrate fun and learning and promote the acquisition of skills through engagement and motivation (3, 4): the players are engaged and motivated to achieve specific goals, rather than focusing solely on entertainment (5–7) and, in this way, increase the time spent on training and improve the quality of learning (8). Some neuroimaging studies suggest that flow may be supported by the joint activation of frontoparietal attention networks and reward-related areas, such as the putamen and striatum (76, 77). Theoretically, this neurochemical environment, often associated with increased dopaminergic activity, is thought to provide a favorable state for synaptic plasticity, potentially reinforcing the learning processes required for cognitive recovery (76).

While neurophysiological data on mental effort remain heterogeneous, research suggests that the positive valence of gaming can make intense concentration feel less onerous, potentially reducing the subjective perception of fatigue (76).

This engagement is not just a secondary benefit but a core clinical variable: by increasing the time spent on training and improving the quality of learning (8), SGs aim to overcome the high dropout rates often associated with traditional, repetitive cognitive exercises. To sustain high levels of user interest, these tools frequently incorporate core ludic mechanics such as competitive elements, goal-oriented progression, cooperative tasks, and immediate feedback. Key features of effective SGs include clear rules, precise objectives, dynamism, positive reinforcement, storytelling, a sense of challenge, and continuous feedback to effectively motivate players to achieve their goals (9).

Crucially, in the context of cognitive training, these mechanics serve as a vehicle for “Near Transfer” (improvement in tasks similar to the trained one) and, ideally, “Far Transfer” (the generalization of gains to daily life and academic performance). The effectiveness of this transfer depends on the training “dose” defined by intensity, frequency, and duration and the degree of task adaptivity, which prevents the user from reaching a performance plateau (78).

In the present study we review studies on SGs to evaluate their effectiveness for attention and executive function (EF) training. For the purpose of this systematic review, we have operationalized SGs based on their primary design purpose and clinical utility rather than simply their technological format. Although SGs represent a specialized subset of digital games, they are characterized by their primary goal-oriented nature: their core design is not driven by entertainment, but by the pursuit of explicit educational or therapeutic objectives (3). In SGs, ludic mechanics, such as rules, goals, and feedback, are explicitly subordinate to a rehabilitative goal (3). Conversely, we excluded commercial video games that were primarily developed for entertainment, even if they might have secondary cognitive benefits. Our focus is on tools where the ludic effort is systematically channeled toward educational or rehabilitative outcomes. Recent studies have demonstrated the effectiveness of SGs in improving cognitive abilities, academic performance (8) and emotional skills (10, 79) in clinical and rehabilitation settings for both pediatric participants (9, 11) and adults (12). SGs are effective rehabilitative tools for individuals with atypical development, such as autism spectrum disorder (ASD) or Intellectual disabilities (13, 14), specific learning disorders [SLD (9)] and attention deficit hyperactivity disorder [ADHD (15)], improving attention, EFs, and social communication skills (16). They usually also increase therapeutic compliance, adherence to treatment, and engagement (17).

A significant contribution to this field is the work of Alopoudi et al. (18), who introduced the “Game4CoSkills” protocol. This initiative moves beyond traditional interventions focused on single deficits. Instead, it proposes an integrated system to simultaneously enhance eight cognitive skills, including logic, multitasking, and visual dexterity. The study targets adult populations that are frequently overlooked, such as those with Intellectual Disabilities, Down Syndrome, and Autism. The protocol follows a mixed-methodology approach, and it includes software development based on clinical needs: a six-month experimental phase with a control group, and qualitative evaluation from caregivers and therapists. The primary goals were to create a mobile SG for Intellectual Disabilities groups and validate its content via neuropsychological testing. Although long-term efficacy and cross-country standardization remain challenges, this approach suggests that mobile tools can mitigate cognitive difficulties and act as a catalyst for social inclusion.

In children with ADHD, recent systematic reviews highlight the potential of SGs to enhance attentional capacities (80). These digital experiences utilize structured rules, feedback loops, and interactive design to foster high levels of engagement and minimize task-related boredom (81). Specifically, interventions focusing on cognitive flexibility and inhibitory control reduce their impulsive behaviors and inhibit inappropriate motor responses (82). However, further research is essential to determine the long-term stability of these improvements (80). Parallel research on ASD suggests that different training modalities may uniquely influence daily functioning. Computerized tools have demonstrated effectiveness in strengthening working memory capacity (WM) and executive attention (83) in ASD. These studies consistently show that SGs can bolster various EF components across different health conditions and age groups, facilitating enhancements in attention and inhibitory control as well as improvements in working memory and cognitive flexibility (73).

The clinical utility of SGs is particularly relevant for the rehabilitation of EFs and attention. EFs are top-down mental processes that allow individuals to monitor and regulate their thoughts and actions in order to facilitate goal-directed behavior. In the field of human development, EFs are viewed as a multidimensional construct primarily composed of three core components: response inhibition, WM, and cognitive flexibility (19). EFs allow individuals to anticipate, plan, set goals, implement goal-directed projects, monitor, and, if necessary, modify their behavior to adapt to new conditions (20). These skills are closely linked to both physical and mental health and well-being, and they are strong predictors of success in human and academic performance and professional life (21–26).

Attention is a multicomponential system, composed of three functionally and neurally distinct networks: the alerting network, the orienting network, and the executive control network (27). The capacity to selectively process stimuli (selective attention) over a prolonged period (sustained attention) is a necessary precursor for more complex cognitive processes (28, 29). In fact, selective and sustained attention play an important role in memory and learning (30). Specifically, suppression-based mechanisms of attentional selection that emerge during the first year of life are critical for cognitive growth (31). In this context, attention and EFs are deeply interconnected: while attention acts as a filter to select relevant information, EFs manage and manipulate that information to achieve complex goals.

SGs have proven useful in improving WM and attention (9, 32). SGs are effective for attentional training because they require players to constantly monitor dynamic environments, ignore distractors, and respond quickly to relevant stimuli. The high frequency of interaction and the requirement for active engagement in SGs improve the neural networks involved in alerting and orienting attentional systems.

However, despite these promising findings, many studies lack the rigor of randomized controlled trials (RCTs), leaving the literature on treatment efficacy somewhat inconclusive. Moreover, the current literature remains fragmented due to notable heterogeneity in sample sizes, participant demographics, and the specific mechanics of the games utilized. Consequently, it remains difficult to determine whether clinical improvements are driven by the training itself or by the motivational appeal of the digital format. Therefore, the primary objective of this systematic review is to evaluate the effectiveness of SGs in rehabilitating attention and EFs in children with both typical and atypical development. Specifically, this work aims to:

1. Identify which cognitive domains (e.g., WM, inhibition) show significant improvement through SGs training;
2. Analyze the efficacy of SGs in improving motivation and engagement;
3. Assess the degree of “far transfer”, i.e., the participant's capacity to apply skills learned through digital training to contexts that are quite different, such as academic and behavioral outcomes.

The systematic review examinee only studies with an untrained control group and pre- and post- training cognitive evaluation of outcomes.

## 2 Materials and methods

### 2.1 Literature search

This study was conducted following the PRISMA guidelines (33) and following PRISMA checklist (33). Specifically, it addressed the research questions, search strategy, inclusion and exclusion criteria, and risk of bias (33, 34). The protocol was not pre-registered. This study was funded by the Italian Ministry of University and Research. Neither the ministry nor any authors have any competing interests.

We identified relevant studies investigating the effectiveness of treatment with SGs in enhancing one or more executive and attentional functions in children and adolescents with typical and atypical development attending primary through high school.

### 2.2 Search strategy and screening process

Web of Science, PubMed, EBSCOhost, and Scopus databases were searched. For EBSCOhost, specific sub-databases were selected to ensure clinical and psychological relevance: APA PsycArticles, APA PsycInfo, eBook Academic Collection (EBSCohost), and Psychology and Behavioral Sciences Collection. For Scopus, the search was implemented using the TITLE-ABS-KEY syntax to target titles, abstracts, and keywords simultaneously. For PubMed, the search was conducted using the [Title/Abstract] tag. For Web of Science, the search was performed using the Topic (TS) field tag, which covers titles, abstracts, and author keywords. The refined records included: Computer Science, Education Educational Research, Engineering, Health Care Sciences Services, Medical Informatics, Psychology, Rehabilitation, Neurosciences Neurology, and Behavioral Sciences.

The search strategy was applied to the fields “Title”, “Abstract”, and “Keywords” (or “Topic” for Web of Science). In the research string, we used three synonyms of SGs, according to the Cochrane Handbook for Systematic Reviews of Interventions (35), to ensure a comprehensive capture of relevant literature. The following string was utilized:

(“serious game*” OR “edugame*” OR “digital game*”) AND (“executive function*” OR “dysexecutive*” OR attention* OR plan* OR inhibition* OR “working memory” OR “cognitive* processes” OR “cognitive flexibility” OR “self-control*” OR metacognit*). Once we identified the relevant articles from these studies, we used the backward reference searching method (the works cited in the selected articles) and a posteriori to further studies (the studies that cited the articles we considered). Moreover, we manually screened the bibliographies of the major systematic reviews and meta-analyses on this topic.

### 2.3 Eligibility criteria

The eligibility criteria were as follows: 1) studies published from January 1, 2014 to May 15, 2024; 2) studies written in English or Italian; 3) studies on human participants; 4) studies providing a SGs intervention.

The inclusion and exclusion criteria were structured according to the PICOS framework [Population, Intervention, Comparison, Outcomes, and Study Design; (84)].

#### 2.3.1 Inclusion criteria

Studies were included in the current review if the following criteria were satisfied:

- Population: Children and adolescents attending school, from the first grade of primary school through the last year of high school (typical and atypical development).
- Intervention: Use of SGs specifically designed and used for training/rehabilitative purposes.
- Comparison: Presence of at least one control group (active or passive/untrained).
- Outcomes: Assessment of at least one core skill of EFs (Inhibition, Working Memory, Cognitive Flexibility) or attentional networks (Selective, Sustained, or Executive Attention) through pre- and post- intervention measures.
- Study design: Experimental designs including both Randomized Controlled Trials (RCTs) and non-randomized controlled studies, provided they included at least one control group (active or passive) with a pre-post assessment.

#### 2.3.2 Exclusion criteria

Studies that met any of the following criteria were excluded:

- Population: Participants outside the school-age range (e.g., preschool or adults).
- Intervention: Use of technological tools other than SGs (e.g., commercial video-games not adapted for training, robots, augmented reality without SG mechanics, Exergames).
- Comparison: Lack of a control group (i.e., groups that received a different treatment or no treatment).
- Outcomes: Lack of assessment of pre- and post- intervention outcomes.
- Study Design: Single cases, reviews, meta-analyses, commentaries, editorials, dissertations, book chapters, or theoretical/qualitative studies. Abstracts-only or posters from conferences were excluded, while full-peer-reviewed conference articles were included.
- Additional criteria for exclusion: duplicated studies; SG not used as an intervention tool, but for other purposes (for example, as an assessment tool); full-text not available; languages other than English or Italian.

#### 2.3.3 Study selection and data extraction

The screening process was performed using the digital tool Rayyan (36). The study selection process consisted of three phases: eliminating duplicates, screening titles and abstracts, and reading full texts. Initially, 6,011 studies were obtained from four scientific databases (Pubmed, Scopus, EBSCO and Web of Science); after removing the duplicates (2,286) all identified records (*N* = 3,725) were screened for potential relevance based on the titles and abstracts by three authors (VT, FB and CR), that independently reviewed the studies for eligibility according to the inclusion/exclusion criteria.

Following this initial screening, the full texts of the potentially eligible articles were downloaded for a comprehensive review. This initial screening phase was performed independently by three reviewers (VT, FB, and CR), who masked their decisions to ensure unbiased eligibility assessment. Any disagreements between reviewers during this phase were resolved through discussion and consensus with a fourth senior reviewer (CVM).

The screening process resulted in the exclusion of 3,495 articles, of which 1,704 articles were rejected due to their type (i.e., systematic, narrative and literature review, overview, meta-analyses, systematic mapping studies, book chapters, books, qualitative studies, case reports, commentaries, theses and conferences). The remaining 1,791 exclusions were based on the outcome of the intervention as the studies did not pertain to the specific areas of interest. These papers focused on topics such as cancer, nutrition, education, animal models, energy, social and naturalistic emergencies and neurodegenerative disorders. Additionally, studies were excluded if they used exergames or targeted the rehabilitation of attention and EFs, the rehabilitation of emotional control, motivation or social skills.

Following this initial screening, the full texts of the potentially eligible articles were downloaded for a comprehensive review to confirm final eligibility. Data extraction was performed independently by two reviewers (VT and FB) using a standardized digital extraction form to ensure systematicity and minimize errors. Inter-rater agreement was very high (99.73% of observations; Cohen's K = 0.53). Any disagreements between reviewers during this phase were resolved through discussion and consensus with a fourth senior reviewer (CVM).

For each included study, the following variables were extracted: (i) participant characteristics (age, population type, diagnosis, and sample size); (ii) presence and type of control groups (active vs. passive); (iii) intervention parameters (SG type, duration, frequency, and total dose); (iv) outcome measures (cognitive tests, clinical scales); and (v) main findings. In case of missing data or discrepancies, the authors of the original studies were contacted, or the issue was resolved through internal consultation between authors.

**Figure 1:**
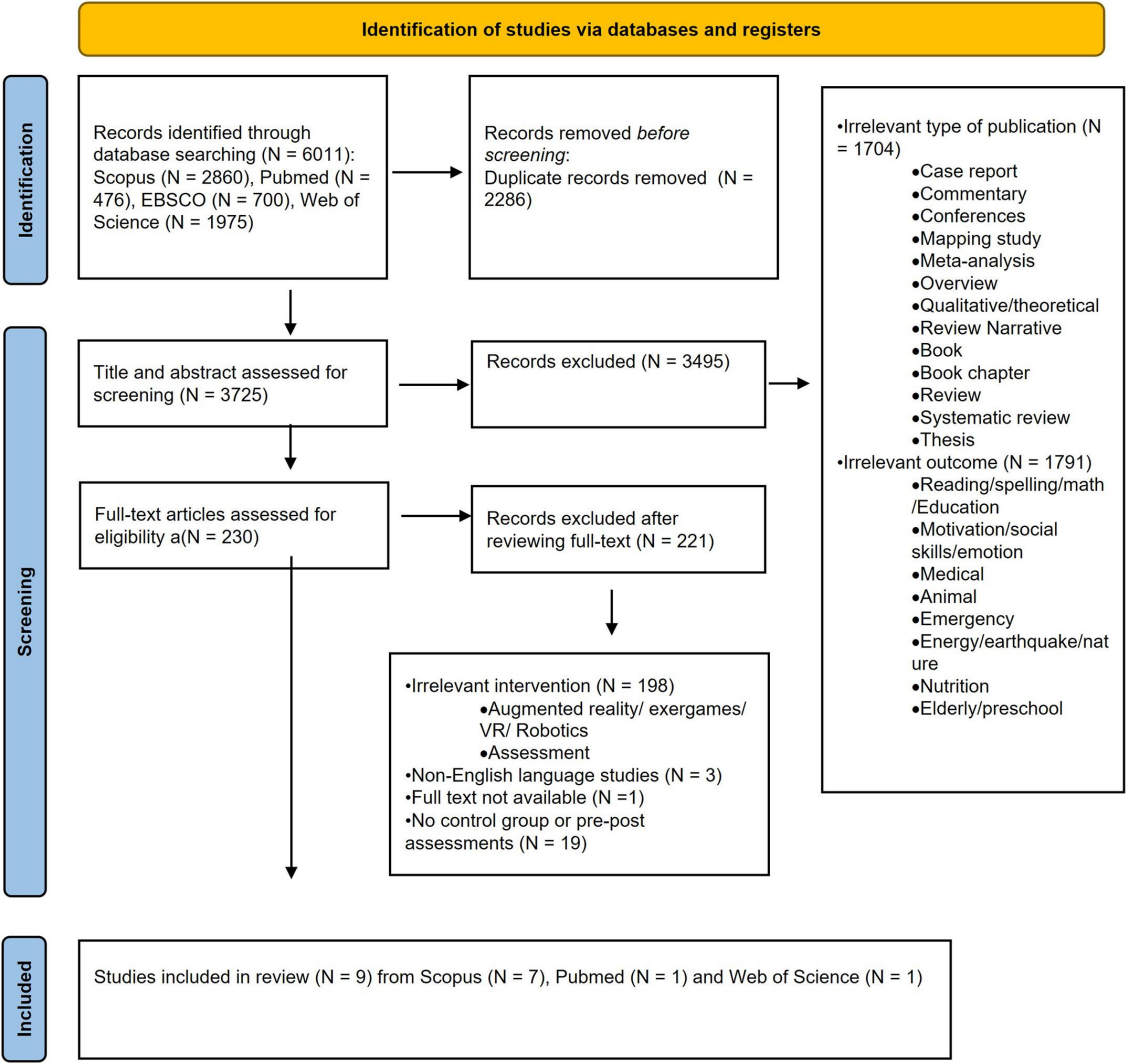
PRISMA flowchart of the literature search and screening process.

Consequently, 230 articles were downloaded in full text for further screening; one study was specifically excluded because the full-text was unavailable. Of these, 220 were excluded after reviewing their full text for various reasons. Many of these studies focused on the use of robotics or augmented reality, while others were excluded because their primary objective was either the creation of a tool or the validation of an assessment scale. Within this final selection process, 9 studies satisfy criteria for inclusion in the systematic review. The results of the screening process are shown in Figure 1.

#### 2.3.4 Risk of bias

The revised Cochrane risk-of-bias tool for randomized trials [RoB 2 (37); Higgins et al., (85)] was used to assess the study's quality and risk of bias. RoB 2 included five domains that might affect the results: The evaluated domains included randomization process (D1), deviations from intended interventions (D2), missing outcome data (D3), measurement of the outcome (D4), and selection of the reported result (D5) (85).

Two authors (VT and VS) independently assessed the methodological quality of the studies using the RoB 2 tool, obtaining a percentage of agreement P(a) = 96% and a Cohen's K of 0.90, i.e., an almost perfect agreement level (38). Furthermore, although there was a strong agreement, any differences were objectively resolved after a discussion with the last author (CVM).

**Figure 2:**
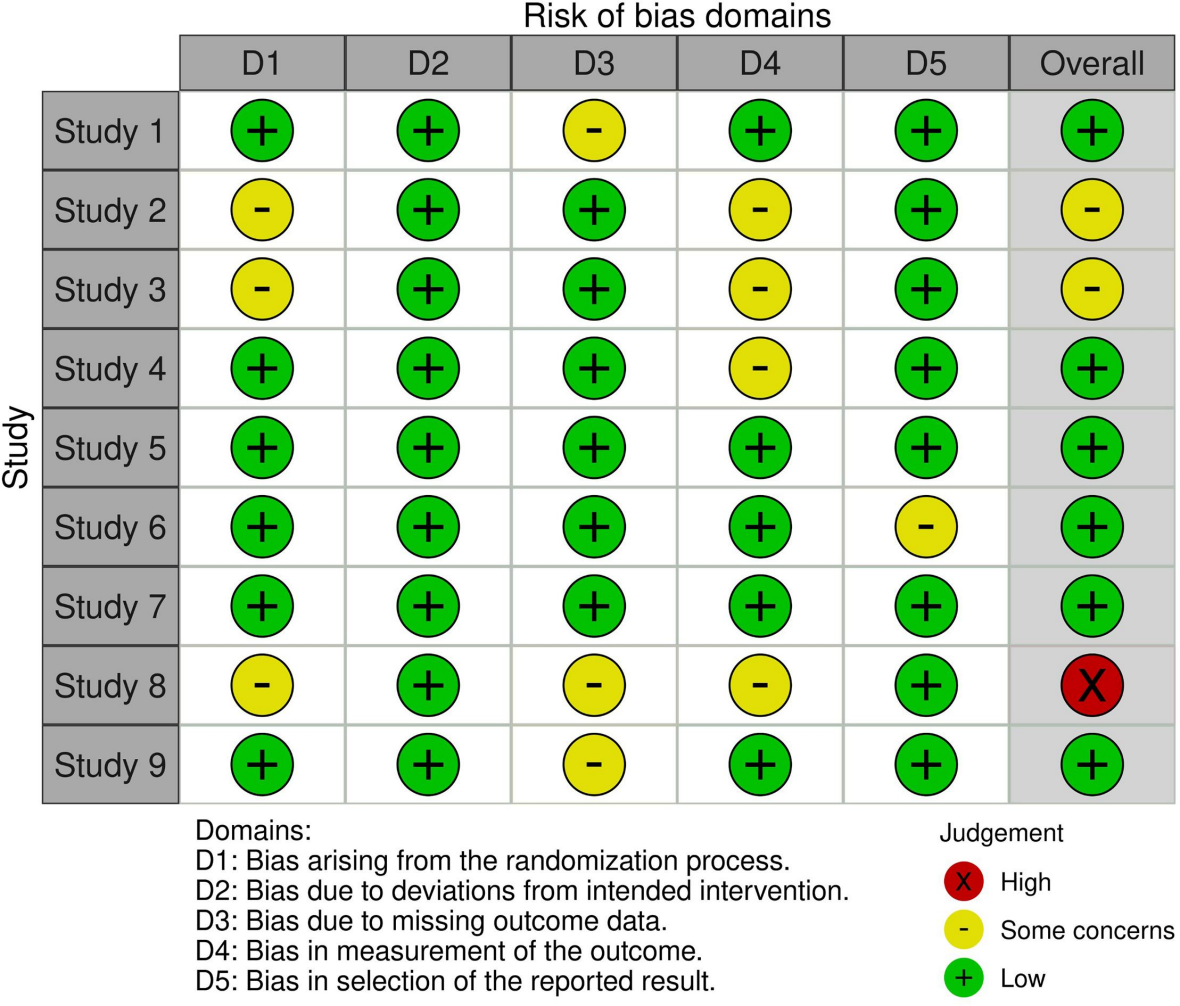
Risk of bias assessment.

The results of each study included in this review are summarized in Figure 2. Overall, the majority of the nine studies demonstrated a low risk of bias. Specifically, Studies 1, 4, 5, 6, 7, and 9 were rated as having a low overall risk, reflecting robust methodological quality across most evaluated domains.

Studies 2 and 3 were identified as having “some concerns” regarding their overall risk. This was primarily due to the use of alternate assignment rather than true randomization, alongside the potential influence of children's specific interest in SGs (D1), and regarding parental supervision during treatment and the explicit admission of potential bias within the study's own limitations section (D3). Study 8 was the only study identified with a high overall risk of bias. The cumulative effect of multiple limitations led to this rating: both the sampling method and the assignment to groups were based on convenience rather than randomized protocols, significant data attrition (D1), with 29 subjects excluded from the final analysis. (D3) and potential for bias due to the teacher's active presence and supervision during the intervention (D4).

## 3 Results

### 3.1 Characteristics of the included studies

Of the 9 articles included in the review, all were published in English from 2014 to 2024 with most articles being published during 2018-2021 (7 studies). Studies were conducted in 5 different countries (Table 1), three of them were multicentric. Regarding the geographical distribution, four studies were conducted in Europe (44.44%), two in Asia (22.22%) and two in South America (22.22%) and one in North America (11.11%). Participants were recruited from various settings: in three cases (Study 1, 2, and 9), recruitment took place across multiple clinical centers or schools located in several suburban areas (Studies 2 and 9). In the remaining studies, participants were recruited either directly from schools (Study 3, 5, 7, and 8) or from clinical centers (Study 4 and 6).

**Table 1:** Characteristics of the studies.

ID	Study	Year	Publication type	Country	Language	Type of design	Pairing
1	Dovis, Oord, Wiers, Prins	2015	Journal article	Netherlands (multicentric study)	Dutch	RCT	Randomization by minimization
2	Gallardo Vergara and Gallardo	2023	Journal article	Cataloni (Spain)	Spanish	RCT	Alternate assignment
3	Ramos and Garcia	2019	Journal article	Brazil	Brazilian	Quasi	N/A
4	Ojani, Kashani-Vahid and Moradi	2020	Conference paper	Teheran (Iran)	Iranian	RCT	Randomization
5	Pashapoor, Kashani-Vahid and Hakimirad	2018	Conference Paper	Rudsar (Iran)	Iranian	RCT	Randomization
6	García-Redondo, Garcia, Areces, Núñez and Rodríguez	2019	Journal article	Northern Spain.	Spanish	RCT	Randomization
7	Boendermaker, Gladwin, Peeters, Prins and Wiers	2018	Journal article	Netherlands	Dutch	RCT	Clustered randomization
8	Ramos and M elo	2019	Journal article	Brazil	Brazilian	Quasi	Convenience Sampling
9	Macoun, Schneider, Bedir, Sheehan and Sung	2021	Journal article	Canada	English	RCT	Randomization

In terms of publication standards, all included papers were peer-reviewed studies, except for Study 4 and Study 5, which were peer-reviewed conference papers. Methodologically, as shown in Table 1, the majority of studies were Randomized Controlled Trials (RCTs) or used a controlled assignment to the experimental or the control group (studies 1, 2, 4, 5, 6, 7 and 9). Specifically, Studies 1 and 7 were classified as RCTs; Study 1 employed randomization by minimization, while Study 7 used a randomized assignment similar to a cluster-based approach. Although Studies 4 and 5 were defined as quasi-experiments, they provided a random assignment to the experimental and control groups. Study 2 utilized an alternating assignment of participants to the two groups. Study 9 was a wait-list control group study with random assignment.

In contrast, Studies 3 and 8 were quasi-experiments: in both studies, the assignment was based on convenience, specifically the accessibility, availability and interest in the technology used. Furthermore, Studies 2, 8, and 9 were identified as pilot studies.

The study populations across all nine investigations were based on convenience sampling; specifically, seven studies involved clinical populations (e.g., ASD, ADHD, SLD), while the remaining two studies utilized cluster-based recruitment within school classrooms. Notably, all identified studies utilized a pre-post- test design, incorporating a control group to evaluate the intervention's impact.

### 3.2 Sample characteristics and demographic information

 Table 2 summarizes some of the demographic and clinical information about the samples examined. Overall, 386 children participated in the selected studies, 36.27% of the participants were females (140) and 63.73% (246) were males. The range of school grades was from first grade to high school. The mean age of participants was 10.18 years (SD = 1.35, range: 6–16 years).

**Table 2:** Characteristics of the population and sample size.

ID	Population	N	N for each Group	Sex	Age Mean	SD	Grade or age range	Pre-test (*N*)		Post-test (*N*)	
								CG	EG	CG	EG
1	ADHD (Combined-type)	89	EG1 29 (Full); EG2 30 (Partial); CG 30 (Placebo)	18F - 71M	10.47	1.4	8-12 years	28	58	25	53
2	ADHD	30	EG 15; CG 15	6F - 24M	9.4	0.6	8-10 years	15	15	15	15
3	Inhibitory control difficulty	8	EG 4; CG 4	2F - 6M	9.0	1.4	2°–6° grade	4	4	4	4
4	Externalizing diseases	20	EG 10; CG 10	0F - 20M	9.7	N/A	3°–4° grade	10	10	10	10
5	Intellectual disabilities	20	EG 10; CG 10	7F - 13M	10.9	N/A	4°–5° grade	10	10	10	10
6	ADHD; SLD	44	EG 24; CG 20	17F - 27M	11.83	2.7	from 1° to 2° high school grade	20	24	20	24
7	typical developing children	84	EG 30 (Full); CG1 30 (standard); CC2 24 (Placebo)	50F - 34M	13.7	0.7	High school	54	30	47	29
8	typical developing children	71	EG 30; CG 41	37F - 34M	7.43	0.7	2°–3° grade	41	30	41	30
9	ASD	20	EG 11; CG 9	3F - 17M	9.16	2.0	6-12 years	9	11	9	11

EG, experimental group; CG, control group; SD, standard deviation.

A total of 231 students with atypical development were examined. In particular, students with: a) learning disorders and ADHD were 44 (Study 6; with 20 participants that perform the control condition -control group, CG- and 24 that perform the training with SG -experimental group, EG-, b) only ADHD were 119 (Studies 1 and 2; CG: 45; EG: 74), c) intellectual disability were 20 (Study 5; CG: 10; EG: 10), d) externalizing disease were 20 (Study 4; CG: 10; EG: 10), e) inhibitory control difficulty were 8 (Study 3; CG: 4; EG: 4), f) ASD were 20 (Study 9; CG: 9; EG: 11). Additionally, 155 typically developing children without any difficulties or disorders were included (Studies 7 and 8; CG: 95; EG: 60).

### 3.3 Characteristics of CG and SG training

In the majority of the studies (Studies 3, 4, 5, 6, 8 and 9), CG did not receive any specific treatment, maintaining their normal routine. In contrast, Study 1 implemented a Placebo condition, where participants used a version of the play lacking cognitive training (in which all tasks were presented in non-adaptive mode, with no stop-signals or rule-switches). In Study 2, the CG performed the same activity of EG, but via traditional paper-and-pencil methods, to evaluate the efficacy of digital tools against conventional methods. Finally, Study 7 compared the SGs intervention against both a standard protocol (without SG elements) and placebo training version (without SG elements and without adaptive difficulty adjustment) to evaluate the added value of gamification.

The Maghzineh game was utilized in two separate clinical studies to target sustained and selective attention. In particular, 12 games of Maghzineh were used with children with behavioral disorders (Study 4) and 7 games with children with intellectual disabilities (Study 5). The Brain School (Escola do Cérebro) program was the focus of two additional studies, though different versions of the platform were implemented, reflecting the evolution of the software. The 2018 intervention (Study 8) featured a suite of five games focused on initial cognitive training protocols. Subsequently, the 2019 intervention (Study 3) utilized an expanded version featuring seven games to improve inhibitory control, EFs and attention in atypical (Study 3) and typical development population (Study 8). Other SGs used across the included studies are: Braingames Brian, SG based on Orjales program, Boogies academy/Cuibrain, City Builder, and Caribbean Quest.

### 3.4 The procedure of administration of SGs training

The procedures used for SGs training are reported separately for each study in Table 3. Training with SG was performed for an average of 5.94 weeks (SD = 3.41; range 2–14), with a mean of 4 weekly sessions (SD = 1.506; range: 2-6) of 30.00 min each (SD = 15.58; range: 10–50).

**Table 3:** Characteristics of CG and EG treatments.

ID	SG Name	Platform	Place of intervention	Supervisors	Period (weeks)	Duration in Hours	Duration in Sessions	Freq. (session per week)	Time of a sessions (in minutes)	CG treatment
1	Braingame Brian (BGB)	Computer	Home	Coach (weekly calls)	5	14-20	25	5	35-50	Placebo (no EFs trained)
2	Serious Game (Orjale’ program)	Computer	Laboratory/School/Clinic	Therapist	4/5	4-7	9	2	30-45	Pencil and paper
3	Brain School	Tablet	Home	Parents	5	3.75	15	3	15	No intervention
4	Maghzineh	Tablet/smartphone	Home	Researcher/mothers	5	N/A	25	5	N/A	No intervention
5	Maghzineh	Website/App	School	Researcher	4	10-15	20	5	30-45	No intervention
6	Boogies academy/ Cuibrain	Tablet/smartphone	Clinic center	Researcher	14	5.07	28	2	10	No intervention
7	City builder	Computer	School	Researcher	2	5	10	5	30	Standard (no game); Placebo (no game, no adaptability of difficulty)
8	Escola do Cérebro (Brain school)	Tablet	School	Researcher/Teacher	6	7.5	36	6	15	No intervention
9	Caribbean Quest (ibrid method)	Laptop	School	Researcher	8	12	24	3	30	No intervention

As shown in Table 3, the game platform for most of the studies included in this systematic review was Personnel Computer (PCs) (*n* = 44.44%) and mobile or tablet applications (*n* = 55.56%). Regarding the intervention setting, the training was conducted at home in three studies (Studies 1, 3, and 4) and within school environments in four cases (Studies 5, 7, 8, and 9). One intervention took place in a clinical center (Study 6), while another was carried out across multiple settings, including a laboratory, school, and clinic (Study 2).

Supervision protocols varied across the studies: in six cases, the training was overseen by a trained professional (a researcher in Studies 5, 6, 7, 9; a therapist in Study 2; and a coach in Study 1). Other studies involved collaborative supervision between researchers and teachers (Study 8) or researchers and mothers (Study 4). In the final case (Study 3), the training was conducted under parental supervision.

### 3.5 Engagement

The analyzed studies demonstrate that gamification is a primary driver for engagement and treatment adherence. However, the methodology for assessing these parameters varies significantly across studies, ranging from direct quantitative metrics to indirect clinical inferences.

Study 1 represents the most significant empirical success in measuring involvement, reporting a motivation and engagement rate of 97% among participants who used the SG, with only a 3% failure rate in program completion. This evidence is similar to that of Study 9 which used qualitative assessments, such as exit interviews with participating parents (70%) and teachers (90%) to confirm that children enjoyed the intervention.

In contrast, Studies 2, 3, 4, 5, 6 and 8 did not formally evaluate engagement through quantitative scales, but authors inferred engagement through the children's constant participation in the training and the inherent appeal of the SG format. For instance, Study 2 inferred engagement by observing significant improvements and the stability of these gains as well as the low rate of dropout (3%) at follow-up, suggesting that the digital format sustained the children's focus more effectively than traditional methods. In Study 3, children's interest in SG was a selection criterion for sample inclusion and a parameter for monitor activities participation. Study 4 employed technical tools for remote performance monitoring to ensure treatment adherence. In Study 5, high motivation was inferred by the successful completion of 20 sessions by children with intellectual disabilities, a population usually lacking curiosity and activity. Consequently, their assiduous participation was viewed by authors as a clear indicator of engagement. Similarly, Study 6 assessed involvement by registering the frequency of attendance at each session. Study 8 allowed children to select specific games during the final week of the intervention based on their personal interests, using this autonomy as a proxy for engagement.

However, a particularly noteworthy counter-trend result appears in Study 7, where enjoyment and motivation decreased in the gamified version. In this specific protocol, children were allowed to perform extra game sessions to earn additional bonuses, yet the number of voluntary exercises chosen by the participants decreased progressively from the second session onward across all conditions, placebo, standard and gamified.

### 3.6 Outcome measures and cognitive rehabilitation targets

Common outcome measures included standardized neuropsychological tests and behavioral questionnaires, although assessment batteries varied significantly across studies. Regarding pre- and post- intervention evaluations, selective and sustained attention were assessed in seven studies (Studies 2, 3, 4, 5, 6, 8, 9). Further assessments were conducted for cognitive flexibility (Studies 3, 5 and 9), inhibitory control (Studies 2, 3, 4, and 5), WM (Studies 3, and 9) and reasoning (Study 3). Notably, Study 1 employed a comprehensive evaluation of selective attention, inhibition, interference control, cognitive flexibility, verbal and visuo-spatial WM, short-term memory and reasoning, while Study 7 maintained a specialized focus exclusively on WM performance.

A distinction must be made between the assessment batteries and the specific cognitive functions targeted for rehabilitation by the SGs. It is worth noting that while all examined SGs aimed to enhance EFs, it is rare to find a SG that strengthens all EFs jointly. Selective and sustained attention were the primary targets for training in seven studies (Studies 2, 3, 4, 5, 6, 8, and 9). Within these interventions, additional rehabilitative modules were included for inhibitory control (Studies 2, 3, 4, 5 and 9), interference control (study 4), and cognitive flexibility (Study 3, 5 and 9), WM (Studies 2, 3, 4, 5, 6, 8 and 9) and planning (Studies 2 and 8). Study 1 implemented a multi-domain training targeting WM, inhibitory control, and cognitive flexibility, short term memory, whereas Study 7 was focused on the rehabilitation of WM. This heterogeneity in training targets reflects the diversity of the SGs’ designs, which range from specialized single-domain tools to complex multi-domain rehabilitative platforms. A summary is presented in Table 4.

**Table 4:** Cognitive functions trained and results. .

ID	Cognitive Skill trained by SGs	Evaluated Skills (pre-post training)	EG gain=CG gain	EG gain > CG gain	Follow up
1	Inhibition; Cognitive flexibility; Visuo-spatial and verbal WM and STM	Selective Att.; Inhibition; Interference control; Cognitive flexibility; Verbal and Visuo-spatial WM; Verbal and visuo-spatial STM; Non-verbal reasoning;	Cognitive flexibility; Verbal WM and STM; Non-verbal reasoning	Selective Att.; Inhibition; Interference control; Visuo-spatial WM and STM	+ 3 months
2	Selective and Sustained Att.; Inhibition; WM; Planning	Selective and Sustained Att.; Inhibition	Selective and Sustained Att.	Inhibition	N/A
3	Selective and Sustained Att.; Inhibition; Cognitive flexibility; Visuo-spatial and verbal WM	Selective and Sustained Att.; Inhibition; Cognitive flexibility; Visuo-spatial and verbal WM and STM; Non verbal reasoning	Selective Att.	Sustained Att.; Inhibition; Cognitive flexibility; Visuo-spatial and verbal WM and STM; Non-verbal reasoning	N/A
4	Selective and Sustained Att.; Inhibition; Interference Control; WM; STM	Selective and Sustained Att.; Inhibition; Interference control	None	Selective and Sustained Att.; Inhibition; Interference Control	No
5	Selective and Sustained Att.; Inhibition; Cognitive flexibility; Visuo-spatial and verbal WM; STM	Selective and Sustained Att.; Inhibition; Cognitive flexibility	None	Selective and Sustained Att.; Inhibition; Cognitive Flexibility	N/A
6	Selective and Sustained Att.;	Selective and Sustained Att.; ADHD symptoms	ADHD symptoms	Selective and Sustained Att.	No
7	WM	WM	WM	None	N/A
8	Selective and Sustained Att.; WM; Planning; Problem solving	Selective and Sustained Att.;	None	Selective and Sustained Att.;	N/A
9	Selective and Sustained Att.; Inhibition; Cognitive flexibility; Visuo-spatial and verbal WM	Selective and Sustained Att.; Divided attention; Cognitive Flexibility; Visual and verbal WM	Sustained attention; Inhibition; Cognitive Flexibility; Verbal WM	Selective Att.; Visuo-spatial WM	No

EG,  experimental group; CG,  control group; Att., Attention; STM, short term memory.

### 3.7 Quality criteria for SGs

The qualitative characteristics of SGs were assessed according to the criteria reported by Caserman et al. (39), by two independent judges (VT and AS). Their rating reports 100% of agreement. Tables 5, 6 report the “serious” and “gaming” characteristics of SGs used in the examined studies.

**Table 5:** “Serious” and “gaming” characteristics of SGs used in the examined studies. .

	Quality aspects	Description	Study 1	Study 2	Study 3	Study 4	Study 5	Study 6	Study 7	Study 8	Study 9
Serious section											
Characterizing goal	Constant focus on the learning objective	Constant focus on the learning objective	+	+	+	+	+	+	+	+	+
		Playful components must not hinder learning	+	+	+	+	+	+	+	+	+
	Clear goals	The indispensability of the characterizing goal	+	+	+	+	+	+	+	+	+
		Clarity of objectives	+	+	+	+	+	+	+	+	+
		The obligation of the serious section	+	+	+	+	+	+	+	+	+
		Feasibility of task execution	+	+	+	+	+	+	+	+	+
Method	Correctness of the domain expert content	Correctness of learning content	+	+	+	+	+	+	+	+	+
		Correctness of technical language	N/A	+	+	N/A	N/A	-	N/A	N/A	+
		Neutrality concerning irrelevant issues	+	+	N/A	+	+	N/A	+	N/A	N/A
	Appropriate feedback on progress	Feedback on performance and progress	+	+	+	+	+	+	+	+	+
		Visible results	+	+	+	+	+	+	+	+	+
		Multimodal feedback	+	N/A	+	+	+	N/A	-	+	N/A
	Appropriate rewards	Positive reinforcement	+	-	+	+	+	+	+	+	+
Game section											
Quality	Proof of effectiveness and sustainable effects - Achieving the objectives	Achieving the objectives	+	+	+	+	+	+	+	+	+
		Retention of acquired learning	+	-	-	-	-	-	-	-	-
	Awards and ratings	Evaluation of the quality of play through recognition by professionals or other players immersive experience	N/A	-	N/A	+	+	N/A	N/A	N/A	N/A
	Ensure player engagement and experience	Fun and involvement	N/A	-	N/A	+	+	-	-	+	+
		Flow Experience	+	+	+	+	+	+	+	+	+
		Multiplayer experience (physical payer)	N/A	N/A	-	-	-	-	N/A	-	-
Enjoyment	Ensure player engagement and experience	Immersive experience	-	-	-	-	-	-	-	-	-
		Fun and involvement	N/A	N/A	+	+	+	+	+	+	+
		Flow experience	+	+	+	N/A	+	+	+	+	+
		Multiplayer experience (artificial player)	+	-	-	+	+	+	-	-	-
	Ensure flow	Alignment between player skill and sense of challenge	+	+	+	+	+	+	+	+	+
		Adaptation of difficulty level	+	+	+	+	+	+	+	+	+
		Fostering motivation to play	+	N/A	-	+	+	+	+	-	+
		Progression of difficulty level	+	+	+	+	+	-	+	+	+
		Variety of play	+	+	+	+	+	+	-	+	N/A
	Establish an emotional connection	Emotional engagement	-	-	N/A	-	-	N/A	-	N/A	+
	Sense of control	Mastery of the game	-	-	N/A	N/A	N/A	N/A	N/A	N/A	+
	Support social interactions	Selection between single-player and multiplayer	N/A	N/A	-	N/A	N/A	N/A	N/A	-	N/A
	Ensure immersive experience	Multimodal sensory stimulations	+	+	+	+	+	N/A	N/A	+	N/A
		Full participation and involvement	+	+	+	+	+	+	+	+	+
Media presentation	Attractive graphics	Graphics appropriate for the game purpose, application area and target group	+	+	+	+	+	+	+	+	N/A
		Clear and non-distracting interface	+	+	+	+	+	+	+	+	N/A
	Appropriate sound	Appropriate music and sound effects	N/A	+	N/A	+	+	+	N/A	N/A	N/A

**Table 6:** Qualitative criteria for the balancing between the “serious” and “gaming” characteristics of SGs used in the examined studies. + = Characteristic present in the SG; - = Characteristic not present in the SG; N/A = Information not available.

	Quality aspects	Description	Study 1	Study 2	Study 3	Study 4	Study 5	Study 6	Study 7	Study 8	Study 9
Integrated serious part with gameplay	Embedding characterizing goals into the gameplay	The synergy between learning and play	+	+	+	+	+	+	+	+	+
		The accordance between play elements and learning task	+	+	+	+	+	+	+	+	+
	Scientific foundation	Team working on game design	+	+	+	+	+	+	+	+	N/A
		Literature review	+	+	+	+	+	+	+	+	+
Interaction technology	Appropriate interaction technology	Appropriate technological tool of use	+	+	+	+	+	+	+	+	+
	Intuitive game mechanics and natural mapping	Presence of tutorials	+	+	+	+	N/A	+	+	+	+
		Intuitive use of game controls	+	+	+	+	+	+	+	+	N/A
		Adherence to reality	-	-	-	-	-	-	-	-	N/A
	No simplifying of the learning and/or training process due to technical features	Ease of use of the technological tool in achieving the objective	N/A	+	N/A	+	+	N/A	N/A	N/A	N/A
		Impossibility of cheating	N/A	N/A	N/A	+	+	N/A	N/A	N/A	N/A
	Avoid adverse effects	Low risk of stress and mental and physical complications	+	N/A	+	N/A	N/A	+	+	+	N/A
		Absence of technical bugs	N/A	-	N/A	N/A	N/A	N/A	+	N/A	N/A

+  =  characteristic present in the SG; -  =  Characteristic not present in the SG; N/A,  information not available.

The analysis of the quality of the included SGs, conducted using the frameworks of Caserman et al. (39), allows us to outline a detailed profile of the pedagogical effectiveness and playful characteristics of the software used. The integration of positive (+), negative (-) and unavailable (*N*/A) values provides an overview of the design choices and methodological gaps in the current literature.

Most of the analyzed SGs have “serious” characteristics: achieving specific learning outcomes or improving EFs is a priority. Criteria such as “Constant focus on the learning objective”, “Clarity of objectives” and the assurance that “Playful components must not hinder learning” were found positively in almost all studies. This suggests that SGs are generally well-designed to integrate a clear educational or rehabilitative intent, a key aspect of their effectiveness. “Feasibility of task execution” also receives a high percentage of positive ratings, indicating that the proposed activities are usually accessible to the target audience. Despite the solid foundation, critical issues emerge in some areas. The “Correctness of the domain expert content” and “Neutrality concerning irrelevant issues” scores show a variable number of negative and N/A ratings. These negative scores indicate that some SGs may not guarantee rigorous scientific validation of the content or may fail to mitigate distractions or biases, suggesting the need for more intensive involvement of domain experts in the development phase. A particularly critical aspect is the “Retention of acquired learning”, which has been met by only one study (Study 1).

The frequent occurrence of “N/A” for “Correctness of technical language” and other criteria indicates a reporting gap in the reviewed studies. This information gap does not necessarily reflect an intrinsic weakness of the SG, but rather a lack of sufficient information in studies.

With respect to the “Game” section, many SGs successfully incorporate key elements of game design for improving engagement. Positive ratings were recorded for “Achieving the objectives” and “Full participation and involvement”: often games guide the user toward task completion and foster active engagement. Difficulty level progression and difficulty level adaptation are also frequently present to keep players challenged and motivated.

There are gaps in the integration of some game elements: Negative or N/A ratings are recorded for features such as “Establish an emotional connection”, “Sense of control”, and “Support social interactions”. Furthermore, the presence of “N/A” in aspects such as “Appropriate music and sound effects”, “Variety of play”, “Establish an emotional connection”, “Sense of control”, “Support social interactions” and “Ensure immersive experience” further indicates a lack of reporting. The lack of information on these aspects prevents a full assessment of the quality of the gaming experience and, consequently, the potential impact on the engagement and motivation necessary for EF training.

Table 6 focuses on qualitative criteria that balance the “serious” and “gaming” components in SGs. Criteria such as “Graphics appropriate for the game purpose, application area, and target group” and “Clear and non-distracting interface” show a high frequency of positive evaluation. The presence of “N/A” in several criteria in this table, such as “Appropriate sound, Appropriate music, and sound effects”, reiterates the problem of incomplete reporting in the SG description.

### 3.8 Evidence of efficacy

In the following section, the efficacy of SGs in improving attention and EFs was examined separately for each developmental population. Table 4 reports the main findings of the studies. As reported above, note that not all EFs were trained simultaneously in any SG.

#### 3.8.1 ADHD and comorbidity SLD

Study 1 examined the improvement of EFs in children with ADHD through the “Braingame Brian” SGs, via a randomized double-blind, placebo-controlled experiment. Participants were divided into three groups: a) a fully active condition, in which visuospatial WM, cognitive flexibility, and response inhibition were trained; b) a partially active condition, in which only inhibition and cognitive flexibility were trained, while the WM task was presented in a non-adaptive placebo-mode; and c) a placebo condition, in which all tasks were presented in placebo-mode (non-adaptive, with no stop-signals or rule-switches). The training consisted of 25 sessions over 5 weeks conducted at home with the support of a telephone coach. Three tasks were carried out: a visuo-spatial WM task in which participants had to remember sequences of positions on a grid; a stop-signals (an inhibition task) requiring to stop when a signal appeared; and a rule-shifting object-sorting task (a cognitive flexibility task) in which they had to change the classification rules of objects. Pre- and post- test measures were assessed using cognitive tasks as well as behavioral evaluations by parents and teachers.

The results emerging from the comparisons between initial and final evaluations highlighted interesting dynamics. Specifically, the improvements in visuospatial WM were strictly limited to the specific task practiced. Significant progress in this skill occurred only in the full-active group, suggesting that WM does not improve through indirect training of other functions, but requires targeted exercise.

Conversely, inhibitory and interference control increased in both Full-Active and Partially-Active groups. An unexpected finding concerned cognitive flexibility, which showed no significant improvements in any of the groups involved, despite being one of the EFs explicitly trained by the SG.

On a behavioral level, the study led to more nuanced conclusions. Although teachers reported improvements in ADHD symptoms, this effect was also observed in the placebo group, making it non-specific. Specifically, when looking at ADHD symptoms and BRIEF [Behavior Rating Inventory of Executive Function (40)] scores, improvements were observed across all three groups (Full-Active, Partially-Active, and Placebo). The BRIEF is a standardized assessment designed to evaluate EFs behaviors in real-world settings (such as home and school), providing a measure of how cognitive deficits manifest in daily life. Because these improvements occurred regardless of the active training content, they are statistically defined as non-specific effects. Furthermore, the gain in the placebo group could depend on non-specific factors (e.g., parent-child interaction or expectations) or from the fact that even the placebo condition requires long-term focus attention.

Despite the high compliance (97%) and the children's motivation to complete the sessions, no significant differential changes were found between the groups in daily executive behaviors or motivation reported by parents via the BRIEF scale. The lack of a significant “Treatment  ×  Time” interaction confirms that active training was no more effective than the placebo in modifying real-world behavior. The study argued that while gamified EF training effectively improves the specific cognitive skills targeted (near transfer), these gains do not necessarily translate into clinical improvements in ADHD symptoms or daily functioning (far transfer).

Study 2 tested improving in selective and sustained attention and inhibitory control in children with ADHD using the SG “Monkey Game” over nine sessions (including exercises aimed at training attention, WM, inhibition and impulse control, visual memory, and planning). The CG completed the same exercises in a traditional paper-and-pencil format. General improvements in selective attention and concentration were observed in both groups (*p* < 0.001), demonstrating that both formats are effective. However, the EG showed a significantly greater reduction in impulsivity compared to the CG. This result indicates that the SG was significantly more effective at improving inhibitory control and motor impulsivity than the traditional pencil-and-paper method.

Only one study (Study 6) examined the efficacy of SGs in improving attention in children with ADHD and SLD. In particular, the SGs “Boogies Academy” and “Cuibrain” have been used. The two SGs were developed using the Tree of Intelligence method, and then Gardner's theory of multiple intelligence (41, 86) was integrated with the game design. The training consisted of 28 sessions, during which the SGs stimulated various intelligences, including logical-mathematical, spatial, linguistic, and others.

Pre- and post- test measures were conducted using a test of selective attention and concentration [D2 Test (42)] and family reports of ADHD symptoms [EDAH Scale (43)]. D2 test results showed significant improvements in attention in the EG. Specifically, the EG demonstrated better attention quality, concentration, and accuracy compared to the control group. No statistically significant differences between the groups were found in the EDAH scale scores. Families did not report a clinical reduction in ADHD symptoms after the intervention. The study suggests that the use of SGs can be integrated into standard treatments to enhance attentional skills, even in children with ADHD and SLD. Although SGs allow for an increase in attention skills in standardized tests, a clinical reduction in ADHD symptoms is not found.

#### 3.8.2 Inhibitory difficulties and externalizing disorders

Study 3 examined the effectiveness of SGs in improving inhibitory control in children identified by teachers and therapists as having specific difficulties in this area. The EG was trained using “Brain School”, a SG platform featuring seven targeted cognitive games designed to rehabilitate several EFs, including sustained and selective attention, cognitive flexibility, WM, inhibition and self-control. The SG included playful activities that incorporated music and body movements.

In contrast, the CG did not have access to the “Brain School” games during the intervention period. The EG demonstrated significant improvements compared to the CG across all assessed functions, showing clear progress in WM and attention, processing speed and cognitive flexibility, and impulse control (inhibition). They exhibited greater accuracy, better self-regulation, and enhanced ability to adapt during playful activities.

Study 4 analyzed the effectiveness of the “Maghzineh” SG to improve response inhibition in children with externalizing emotional-behavioral disorders. These disorders include issues such as impulsivity, hyperactivity, and difficulty in behavior regulation, making response inhibition a crucial skill for managing impulsive reactions. The EG engaged in 24 training sessions over five weeks using the Maghzineh Attention Package. The intervention consisted of 12 specific tasks designed to rehabilitate response inhibition, selective and sustained attention and WM. In contrast, the CG did not receive any cognitive intervention during the study period and was placed on a waiting list. Participants were screened before and after the training with the Child Behavior Checklist [CBCL (44)] and the Stroop Color and Word Test [SCWT (45)], assessing response inhibition. Results showed larger improvement in inhibition control in the EG compared to CG in the post- test phase. Additionally, results highlighted a significant enhancement in selective and sustained attention: they improved attentional filtering and allowed them to remain focused on tasks, which directly correlated with their increased scores in accuracy and overall cognitive control. This study suggested that Maghzineh SG is an effective tool for improving response inhibition in children with externalizing disorders.

#### 3.8.3 Intellectual disabilities

Study 5 examined the effectiveness of SGs in improving attentional skills in students with intellectual disabilities. These students often struggle with attention, concentration, WM, learning, and social and behavioral adaptability (46). The study explored whether a SG can genuinely enhance their attention by reducing errors and improving response times. The EG underwent training on the “Maghzineh” SG, which included seven targeted cognitive games designed to increase selective and sustained attention, verbal and visuo-spatial WM and inhibition control, reduce interference, and enhance memory and processing speed.

Pre- and post- test measures were conducted using the Stroop Color-Word Test (45) to assess response inhibition, attention, and cognitive flexibility. The results showed a significant larger gain in the EG compared to the CG, with an improvement in selective and sustained attention and response inhibition (errors and time reduction in the Stroop test). The repeated practice and hand-eye coordination required by the SG effectively boosted WM capacity and the students’ ability to adapt their responses to interfering stimuli. No changes were observed in the CG. These findings suggest that SGs can enhance attention and the ability to inhibit automatic and interfering responses in children with intellectual disabilities.

#### 3.8.4 Autism Spectrum disorder

Study 9 investigated the efficacy of “Caribbean Quest”, a SG designed to improve attention and EFs in children with ASD. Delivered in a school setting, the study aimed to evaluate both near-transfer (improvements in objective cognitive performance) and far transfer (gain academic achievement) effects. Caribbean Quest is a SG that uses a hybrid approach, combining process-specific training with compensatory strategies (metacognitive coaching). The participating group performed the intervention in a one-to-one format during school hours (3 sessions/week, 30 min each, totaling 12 h over 8–10 weeks), while the CG was placed on a waitlist and began the training after the EG.

The EG showed a significant reduction in errors compared to the CG on the KiTAP Sad/Happy Ghost task (47), measuring selective attention. A significant reduction of errors was found in the Colored Boxes task (48), assessing visuo-spatial WM. No significant gains were found in verbal WM, sustained attention, and cognitive flexibility.

Regarding far transfer, the EG showed a significant reduction in the proportion of errors in math fluency and in the problems solving. In contrast, no significant improvements were seen for oral reading fluency (note that training's focus primarily on visual processes rather than verbal ones).

#### 3.8.5 Neurotypical developing students

Study 7 investigated whether incorporating game elements into a WM training program could boost and sustain motivation. In this study, students were divided into three groups: 1) In the gamified training group, a WM task was integrated into the SG “City Builder”, and points earned through correct trials allowed players to build a virtual city; 2) The Standard Training group performed the same WM task but without any gaming elements or rewards; 3) For Placebo group a similar activity but without an impact on WM skill were used.

Experimental gamified group engaged in the training for a longer period compared to the other groups, indicating that the SG successfully boosted the motivation to train. However, the motivational effect of the SG was not permanent; it was found to fade over time as the sessions progressed. WM capacity increased equally in all conditions, including the placebo condition: the SG helped sustain motivation during training, but did not enhance cognitive benefits compared to traditional training.

Study 8 investigated whether the daily use of SGs in the classroom can improve attention in primary school children. The EG played the SG on tablets for six weeks, while the CG followed the standard school curriculum. Pre- and post- intervention measures were recorded using a selective attention and concentration test [D2 Attention test (42)].

The results showed that the SGs enhanced students’ attention performance. Attention improvement was partially due to time, as both groups increased their total scores in the final assessment. However, the SGs group showed a greater improvement compared to the CG. This study demonstrated that schools could integrate SGs as a strategy to enhance students’ attention and cognitive development.

### 3.9 Near and far transfer

The transfer of cognitive improvements into functional real-world gain (i.e., far transfer) remains a complex challenge within the analyzed studies. While most studies reported significant “near-transfer” effects (improvements in the specific tasks trained), evidence for “ far transfer” (generalization to daily life or academic performance) varied considerably and was often absent.

The studies analyzed demonstrate that SGs consistently produce significant near transfer effects, specifically improving the cognitive functions directly trained (such as selective and sustained attention, inhibition and WM). However, the evidence for far transfer is more heterogeneous: while some interventions successfully generalized gains to academic skills, like math fluency and classroom behavior, others showed improvements limited to the digital context (and not generalized to scholastic performance) or driven by non-specific motivational factors (rather than intrinsic cognitive changes).

However, it is crucial to emphasize that such generalization did not emerge consistently across all 9 reviewed studies. A representative example is Study 1, which highlights a clear distinction between different types of cognitive gains. Improvements in inhibition and WM were near transfer effects, specifically related to the type of treatment received (full-active training and partially training). In contrast, far transfer changes, such as improvements in untrained EFs (e.g., interference control) and behavior (self-monitoring), were mostly nonspecific, as they also appeared in

placebo condition. This highlights a critical finding: while treated functions (Near Transfer) improve only through specific training, improvements in general behavior (Far Transfer) occur similarly even without actual cognitive training.

In contrast, Study 2 demonstrated that while both SGs and traditional formats improved selective and sustained attention (near transfer), the SGs intervention was uniquely effective in achieving a significant reduction in commission errors (false alarms; inhibition). However, this study did not evaluate far transfer; the authors explicitly noted as a limitation that the measures were confined to attention deficits and did not include behavioral, motivational, or clinical assessments in real-world settings (e.g., classroom or home).

The Study 3 further supports the potential for far transfer by demonstrating that the use of “Brain School” SG led to comprehensive cognitive gains. The participants showed not only near transfer, improvement in the domains directly targeted such as spatial WM, sustained attention inhibition and cognitive flexibility, but also progress in untrained areas including verbal WM, STM and non-verbal reasoning. Furthermore, the study evidenced far transfer through qualitative improvements in self-control and self-regulation, observed during varied playful contexts such as music activities. Interestingly, the researchers noted a reduction in the time required to complete cognitive tasks.

Similarly, Study 4 demonstrated significant near transfer improvements in response inhibition and selective attention for children with externalizing disorders. The researchers noted that these cognitive gains translated into better far transfer outcomes, specifically in abstract problem-solving, cognitive control processes such as decision making and impulse control. Notably, the study emphasizes that this transfer is strictly dependent on the serious nature of the game; unlike commercial video games that may encourage mindless repetition, these targeted cognitive SGs require the active use of EFs to progress, potentially triggering neuroplasticity to support appropriate behavior in real-life.

In the Study 5, the use of the SG with students with intellectual disabilities revealed robust near transfer effects in EFs trained. Authors attributed this success to hand-eye coordination and software-guided repetition that stimulate neural flexibility (synaptic plasticity). However, note that the study provided no empirical evidence of far transfer: the results remain confined to performance in computerized tasks and the stabilization of attention during game sessions, without extending to independent behavioral or academic measures.

Study 6 shows a marked discrepancy between different types of transfer. On one hand, clear near transfer was observed in sustained and selective attention. On the other hand, the study failed to find evidence of far transfer to daily life: family reports [EDAH (49)] showed no significant reduction in clinical ADHD symptoms.

Results of Study 7, focused on WM training, showed a clear near transfer effect in training engagement: the gamified group performed significantly more bonus trials, demonstrating that the game elements successfully boosted the training motivation. However, the study did not find specific superior cognitive near transfer (WM improved across all groups due to practice effects). Regarding far transfer, the intervention produced no changes in alcohol consumption behavior, although baseline consumption levels were already low. However, a gamified environment decreased motivation to perform well on the post- test assessment, likely because the rewarding nature of the game made the standard assessment tedious.

Study 8 investigated the effects of a 15-minute daily digital game routine within a real school environment. The results showed a strong near transfer effect according to which EG exhibited a significantly higher improvement in selective and sustained attention (near transfer). Regarding far transfer, the authors suggest that the improvement in attention span may not be intrinsically tied to the games’ features, but rather to behavioral training facilitated by the context.

Study 9 examined a hybrid intervention (use of SGs combined with adult-led coaching) for children with ASD. The results indicated clear near transfer in selective attention and visual WM. Crucially, the study provides evidence of far transfer to academic achievement, specifically in math fluency. The intervention group demonstrated a significant reduction in the proportion of math errors Qualitative data further supported far transfer, with teachers reporting an increase in unprompted metacognitive strategy use (e.g., repeating instructions aloud) and improved self-regulation in the classroom. This success is attributed to the hybrid model, which combined the motivational SGs format with adult-led coaching, to bridge the gap between digital play and real-world application.

### 3.10 Brief summary of the results

 Table 7 reports a brief summary of the results of examined studies, highlighting a predominance of near-transfer effects and a significant reliance on indirect measures for engagement. The most consistent positive results are observed in Inhibitory Control. Out of the six studies that investigated this domain, five reported significant improvements (Studies 1, 2, 3, 4, 5), while only one showed no effect (Study 9) and the other three did not investigate or rehabilitate this specific function (Studies 6, 7, 8). Similarly, Interference Control (attentional control) showed success in the two studies (Studies 1 and 4) where it was measured. Results for Selective Attention are varied. Specifically, improvements were observed in Study 1, 4, 5, 6, 8, and 9, while no effect was observed in Study 2, 3, and 7. Sustained Attention was examined in 7 studies: an improvement was observed in Studies 3, 4, 5, 6 and 8, while no effect was found in Study 9. WM shows the most inconsistent results: Study 3 and 5 showed WM improvements, Study 1 showed mixed results, and Study 7 and 9 reported no significant gains. Only Study 3 and 5 reported improvements in cognitive flexibility, Study 1 and 9 did not report gains in this skill, while other studies did not test it.

**Table 7:** Efficacy of SGs training.

ID	Cognitive flexibility/shifting	Inhibitory Control	Interference control (attentional control	Selective Attention	Sustained attention	WM	Engagement	Far transfer
1	-	+	+	+	N/A	+/-	+ (not standardized test)	Non specific
2	N/A	+	N/A	-	-	N/A	N/A	N/A
3	+	+	N/A	-	+	+	+ (indirectly	+
4	N/A	+	+	+	+	N/A	+ (indirectly	+
5	+	+	N/A	+	+	N/A	+ (indirectly	-
6	N/A	N/A	N/A	+	+	N/A	+ (indirectly)	-
7	N/A	N/A	N/A	N/A	N/A	-	-	N/A
8	N/A	N/A	N/A	+	+	N/A	+ (indirectly)	N/A
9	-	-	N/A	+	-	+	+ (not standardized test)	+

Plus (+) indicates a statistical improvement in the group trained with the SGs compared to the control group; Minus (-) indicates similar pattern in experimental and control groups; Plus/minus (+/-) indicate a greater improvement after SGs training but limited only to specific conditions; N/A indicates that the ability was not investigated;+(indirect) indicate indicates that engagement was evaluated indirectly through performance improvement (if performance improves, it is assumed that the participant was actively engaged in the task).

The effectiveness of SGs training appears to be highly population-dependent. While it may serve as a powerful tool for enhancing inhibitory control in ADHD, its impact on the ASD population is much more limited and specialized. A major limitation is the scarcity of literature: for instance, only one study in this review specifically addressed the ASD population, making it difficult to generalize these findings or determine if the failures in rehabilitation depend on the task design or the intrinsic nature of the disorder.

The synthesis of the results reveals that duration and intensity of training have not always been systematically considered in existing literature. In fact, the total number of required training hours across the studies varied from a minimum of 3.75 to a maximum of 20 h. Similarly, session duration ranged from a brief 10-minute engagement to a more intensive 50-minute period, while the frequency of sessions per week also showed considerable variation across the different protocols.

## 4 Discussion

This systematic review analyzed the effectiveness of SGs in training attention and EFs in the school-aged population with typical (2 studies) and atypical development (7 studies). Historically, SGs have been widely used to improve cognitive abilities (50), academic performance (51) and social skills (13, 14), especially in individuals with developmental disorders such as ADHD (16), dyslexia and dysgraphia (9), and Down Syndrome (73).

The current systematic review included studies assessing the effect of SGs on different outcomes such as WM, sustained and focused attention, inhibitory control and cognitive flexibility. The extensive search across multiple databases allowed 6,011 studies to identify. Following a rigorous screening process based on inclusion criteria, specifically a pre-post treatment design with both experimental and control groups, and a focus on school-aged populations, nine studies were selected for final analysis. The risk of bias assessment for the nine included studies was conducted across five main domains, revealing a generally high level of methodological quality. Six articles achieved an overall low risk of bias, demonstrating rigorous procedures from randomization to result reporting. In contrast, two studies raised some concerns primarily due to potential weaknesses in the randomization process and uncertainties in outcome measurement, which may affect the objectivity of their findings. Only one study was classified as high risk (Study 8), largely due to significant issues in the measurement of outcomes and concerns regarding missing data and the randomization process itself. However, the predominance of low-risk studies strengthens the reliability of the findings, suggesting that the observed improvements in cognitive functions are likely attributable to the interventions rather than methodological artifacts. From a methodological perspective, interpreting the effectiveness of SGs requires acknowledging that the findings of this systematic review offer only preliminary support for the potential of SGs to enhance attention and EFs. However, the modest number of included studies (*n* = 9) and the significant heterogeneity across populations (from ADHD children to healthy older adults) limit the results. Main findings are discussed in the function of research questions.

### 4.1 Clinical efficacy in function of the type of population-

The data analysis highlights how the effectiveness of SGs is closely linked to the cognitive architecture of the specific clinical population. The population examined were extremally heterogeneity, ranging from neurotypical children to those with ASD, ADHD, and Intellectual Disabilities. In fact, deficits in EFs and WM represent the common core of all these disorders. In these contexts, traditional rehabilitation often fails due to poor adherence to repetitive tasks, making a game-based approach not just a design choice, but a therapeutic necessity (52). SGs address this gap by providing continuous feedback and difficulty regulation, which are particularly beneficial for populations with a deficit in EFs (87).

The findings of our review and previous reviews were consistent for some outcomes and different for others. Recent literature confirms the general efficacy of SGs. For instance, a systematic review on neurodevelopmental disorders (73) reported significant improvements in EFs such as attention, WM, and cognitive flexibility, particularly in populations with Down syndrome. Similarly, Shahmoradi et al. (52) found that in 19 out of 26 studies analyzed, SGs for attention rehabilitation produced at least one significant improvement, reinforcing the clinical value of these tools for therapists. In this context our study provides a detailed breakdown of specific attentional and inhibitory sub-domains: while Inhibitory Control is highly responsive in ADHD/Externalizing populations (Studies 1, 4, 5), it remains largely resistant in the ASD population (Study 9). The data indicates that SGs training is most effective for inhibitory control and attentional processes, which represent essential core executive abilities. Inhibitory control shows robust and consistent benefits specifically in populations with ADHD and externalizing disorders (Studies 1, 4, 5). In these cases, gamified training acts as a direct reinforcement for response suppression. Conversely, in the ASD population (Study 9), this function appears resistant to training, showing no significant effects. Moreover, for the population with ASD, the only domains showing any degree of success are selective attention and WM (Study 9), while other EFs (inhibition, flexibility, sustained attention) fail to show improvement. This suggests that populations with neurodivergences may require highly specific task designs or that their cognitive profile responds only to certain types of computerized stimuli.

Cognitive Flexibility and WM exhibit much more inconsistent results. These abilities appear more resistant to training, with improvements often limited to specific studies. However, a critical observation is that many of the reviewed studies did not evaluate or target these specific functions at all (represented by the high frequency of N/A values in Table 7). This lack of systematic assessment means that the results for these domains cannot be conclusively discussed in terms of general efficacy. It remains unclear whether the inconsistent findings in literature are due to a genuine resistance to training or simply to a lack of targeted rehabilitative design and objective measurements. Furthermore, the variability in efficacy training for Selective and Sustained Attention suggests that the efficacymay depend heavily on the specific design of the game or the clinical profile of the target population.

Regarding older populations, Wong et al. (53) demonstrated that game mechanics, like difficulty and pressure, significantly impact attention and EFs improvement, while competition appears less influential. Then, the positive effect of SGs on the rehabilitation of EFs is also demonstrated in adults. However, the degree of efficacy in this population is not always consistent: it can be significantly influenced by the presence of pathological aging (such as Mild Cognitive Impairment) or by technical and individual factors (such as medication dosing, emotional control, and the ongoing support from companions for users with limited technological capabilities). This variability may explain why, in some cases involving adults with intellectual disabilities, improvements in domains like planning and decision-making do not always reach statistical significance (54). The present review has demonstrated SGs efficacy for EFs and attentional training also in school age, an age in which SGs can be used not only in the clinical setting but also in the school setting.

Evidence synthesized in this review suggests that SGs are promising tools for improving specific core cognitive processes. This applies across a broad spectrum of neurodevelopmental profiles, although the efficacy to foster generalization to real-world outcomes remains inconsistent. In particular, SGs appear to be highly effective for the rehabilitation of attention among children with intellectual disabilities and behavioral disorders, specifically targeting inhibitory control and externalizing symptoms. The consistency of these findings across multiple studies, many characterized by low risk of bias (e.g., Study 1), supports the potential of SGs as reliable clinical tools for ADHD and comorbid learning profiles. While inhibitory control training appears highly effective for ADHD, its impact on ASD cohorts remains negligible, indicating that the cognitive profile of the clinical population deeply affect the efficacy of gamified interventions.

Positive outcomes were also consistently observed in children with typical development, confirming the versatility of these tools. Regarding ADHD, while general efficacy was noted, the most significant impact was observed in children presenting with comorbid SLD. Furthermore, behavioral inhibition was successfully rehabilitated across most atypical populations; however, an important exception emerged regarding ASD, where inhibitory control did not show the same level of improvement as other executive domains.

Conversely, the reported improvements in attention span for ADHD from Study 8 must be considered with caution. The high risk of bias, particularly regarding missing data and outcome measurement, weakens the reliability of this result and may explain why such gains did not translate into clinical symptom reduction in daily life.

.

### 4.2 Synthesis of cognitive improvements (question 1)

To answer the first research question regarding the most significant cognitive improvements, a key finding is that all nine analyzed studies demonstrated efficacy. Each study successfully rehabilitated at least attention and one additional EF. Specifically, WM was successfully rehabilitated in Study 1, 3 and 9, while Inhibitory Control saw significant improvements in Studies 1, 2, 3, 4, and 5. Furthermore, Interference Control gains were specifically reported in Studies 1 and 4, and Cognitive Flexibility was effectively stimulated in Studies 3 and 5.

Detailed analysis of these gains reveals critical insights into the functional architecture of EFs. For example study 1 reveals interesting dynamics regarding the functional architecture of EFs. The improvements in visuospatial WM were strictly limited to the specific task trained; significant progress in this area occurred only in the full-active condition. This suggests that WM does not improve through indirect training of other functions but requires targeted exercise. Conversely, inhibitory and interference control increased in both Full-Active and Partially-Active groups. This finding is particularly interesting as it suggests that response inhibition and WM are functionally independent. Furthermore, the observed behavioral improvements in all groups suggest that non-specific factors, such as the placebo effect or the positive impact of a gamified routine, play a significant role.

Similarly, results of Study 3 reinforced the potential of these tools for behavioral regulation. Some concerns raised in the bias assessment suggest that the magnitude of these effects should be validated by future high-rigor studies. In this intervention, the experimental group demonstrated significant improvements compared to the control group across all assessed functions. They exhibited greater accuracy and enhanced impulse control during playful activities. Interestingly, the researchers noted a reduction in the time required to complete cognitive tasks, suggesting that SGs may enhance the efficiency of cognitive processing, a critical foundation for academic learning and behavioral stability. This study confirmed that SGs are an effective and motivating strategy capable of improving inhibitory control in children with self-regulation difficulties.

This potential for neurorehabilitation was also evident in Study 5, which focused on children with intellectual disabilities. The results showed a significant improvement in the EG compared to the CG in selective and sustained attention and response inhibition. The findings suggested that the repeated practice and hand-eye coordination required by the SG effectively boosted WM capacity and the students’ ability to adapt their responses to interfering stimuli. These results suggest that SGs can improve the ability to inhibit automatic and interfering responses even in populations with significant cognitive impairments.

Study 9 provided critical evidence of far transfer through a hybrid intervention model for children with ASD. The results indicated that the expansion of executive capacity, in WM and attention, allowed for more accurate mental manipulation of information, leading to a significant reduction in math errors.

Two notable exceptions emerged. Study 2, was only partially successful, showing significant gains only in inhibitory control, despite attempting to target multiple functions. Methodological issues in randomization or measurement might have limited its ability to demonstrate broader cognitive benefits. Similarly, Study 7 failed to show superior efficacy in rehabilitating WM compared to standard or placebo conditions. It is important to note, however, that the primary objective of Study 7 was to verify whether game elements could enhance performance in WM rehabilitation compared to non-game settings, rather than just measuring the rehabilitation itself. In this study, WM capacity increased equally in all conditions, including the placebo condition. This suggests that the SG helped sustain motivation during training, but did not enhance cognitive benefits compared to traditional training. In fact, the experimental gamified group engaged in the training for a longer period compared to the other groups, indicating that the SGs successfully boosted the motivation to train. However, this motivational advantage was not sustained throughout the entire intervention; the effect gradually diminished over time as the training sessions progressed.

Among the SGs used in the studies examined, Maghzineh (Studies 4 and 5), Braingame Brian (Study 1) and Brian School (Study 3) seem particularly versatile, successfully rehabilitating a wide breadth of EFs. In contrast, the intervention for children with ASD in Study 9 was more targeted: it focused on fewer functions, but achieved significant improvements in those specific areas. However, it is important to note that most studies focused on specific EFs, rather than an overall enhancement. The lack of research that trained all EFs within a single SGs remains a barrier to verifying whether a comprehensive digital tool can effectively strengthen the entire profile of attention and executive skills in both typical and atypical students.

### 4.3 Training parameters

The effectiveness of these interventions, however, cannot be interpreted as an absolute value; it depends on precise parameters that must be monitored (78). As established in the cognitive training literature, it is important not only the type of training (SGs vs. paper and pencil rehabilitation), but also reaching the required quantity of training: without a sufficient cognitive load, the neural plasticity processes necessary for stable changes are not activated.

Despite the relatively short duration of the interventions in terms of total weeks (from a min of 2 to max of 14 weeks) and session hours (from a min 10 to a max of 50 min), the EGs consistently showed greater improvements in trained EFs compared to control groups. The analysis of training dosage reveals a non-linear relationship between total training hours and outcomes. As noted by Shawn Green et al. (78), an intervention that works in a laboratory may lose efficacy in the real world due to poor compliance. This explains why studies with lower dosages (e.g., Study 4) might still achieve far-transfer if the engagement is high, whereas more intensive training (e.g., Study 5) may fail if the cognitive load is poorly calibrated or perceived as boring.

A representative model of an effective dosage is often provided by Wexler et al. (88), who suggest that a structured routine (e.g., three 20-minute sessions per week over four months) is key to producing significant gains. However, our review reveals a more complex picture. For instance, Studies 3, 4, and 9 successfully demonstrated far-transfer (the rehabilitation of domains not directly trained). Interestingly, the training quantity varied significantly among them: Study 3 involved 4-7 total hours (30 min, twice a week), Study 4 only about 4 total hours (15 sessions of 15 min), and Study 9 approximately 12 h (24 sessions of 30 min). Conversely, Study 5 (10-15 total hours) and Study 6 (about 5 h) failed to show far transfer effects, while in other studies, this information was not specified.

This discrepancy suggests that the dose is not merely a matter of total duration and frequency. It must be interpreted alongside the Risk of Bias and user engagement. While Studies 4 and 9 achieved far transfer with a Low Risk of Bias, Study 3, which also showed positive results, raised some concerns in the bias assessment. On the other hand, Studies 5 and 6 failed to show generalization despite being Low Risk and, in the case of Study 5, having a high number of training hours (10-15). This indicates that the modality and the level of engagement are decisive: if the child is not motivated, they may perform the task mechanically without the necessary cognitive effort.

All studies primarily utilized laptops or tablets for SGs training, showing no differences in function of the type of tool used. Training is conducted either in home settings or integrated into the school routine with the assistance of researchers or teacher/parents. Also in this case, there does not appear to be any particular difference in effectiveness based on the type of setting.

### 4.4 Motivation and engagement dynamics (question 2)

To answer the second research question concerning the role of motivation and engagement in treatment adherence, this review acknowledges a significant methodological gap: in the majority of the studies analyzed, these constructs were not evaluated through standardized quantitative scales. For six out of nine studies, formal quantitative data on motivation was absent, being present in only three studies. Consequently, the authors inferred engagement indirectly from indicators such as high treatment adherence, the successful completion of even the longest training sessions, and other behavioral cues observed during the interventions. While these behavioral cues are suggestive, they do not constitute a direct psychometric measurement of the constructs, thus requiring caution in interpreting motivation as a proven causal mechanism for success. Nevertheless, the lack of objective and systematic metrics for motivation represents a methodological limitation acknowledged by many authors. Where measured, as in Study 7, it was observed that SGs significantly boosted behavioral motivation through bonus trials. However, this effect tended to fade over time, suggesting that gamification alone may not be sufficient to maintain long-term clinical adherence without constant content updates (see Table 7).

While existing literature often treats motivation and engagement as inherent properties of SGs, our findings suggest that high adherence cannot be guaranteed simply by the digital nature of the intervention. It is often assumed that users are inherently motivated by gaming, but this is not always assessed. Engagement remains a variable that requires active monitoring and structured support. However, studies often make only indirect evidence of engagement and motivation level, without a direct measurement and objective monitoring of this aspect.

In Study 7, the number of voluntary exercises (children were allowed to perform extra game sessions to earn bonuses) were considered a measure of enjoyment in playing the SG. Findings of this study highlight that enjoyment and motivation actually decreased in the gamified version. In this study, the number of voluntary exercises decreased progressively from the second session onward across all conditions (placebo, standard, and gamified). This highlights that the dose is ineffective if the engagement is not sustained. Without active interest, even a high frequency of training fails to trigger lasting rehabilitation.

This suggests that poorly calibrated gamification, or a design perceived as an unnecessary cognitive load, may produce an effect opposite to what was intended. Consistently monitoring adherence and engagement is therefore fundamental for several clinical and methodological reasons. A high level of involvement ensures that the child completes the entire rehabilitative protocol, which often spans several weeks, thereby preventing drop-outs.

Nonetheless, as highlighted by the authors of Study 2, the lack of formal motivational measures remains a significant limitation in several current designs, suggesting that “future research should focus on measuring the level of motivation and treatment adherence” for the SG format to better validate clinical outcomes. This indicates a general awareness among SGs developers of the importance of an adequate visual presentation and an intuitive user interface for the effectiveness of the game. Good usability and relevant aesthetics are crucial for reducing cognitive load and maintaining focus on learning/rehabilitation objectives. While these may seem like details, they significantly contribute to the atmosphere, immersion, and player engagement (9).

The ability to effectively balance the serious and gaming components is crucial for creating effective SGs (9, 39). However, if studies do not report details on how these elements were considered or implemented, it becomes difficult to evaluate the success of this balance and draw robust conclusions about their overall quality. In summary, SGs hold promises for EFs training, but their potential is still limited by the challenges of ensuring detailed data that allow for comprehensive evaluation, replication and optimization of SGs. Finally, the lack of objective metrics for engagement makes it difficult to determine whether adherence was driven by the seriousness of the task or merely by the novelty of the digital format.

There are gaps also in the integration of other game elements that can impact on the overall experience. Negative or N/A ratings are recorded for features such as establish an emotional connection, sense of control, and support social interactions. These elements are recognized in the literature as crucial for promoting deep engagement and intrinsic motivation (55, 56). The absence of a strong emotional connection or sense of control can reduce immersion and player satisfaction, limiting the long-term effectiveness of SG (57, 58).

### 4.5 Mechanisms of Far-transfer and generalization (question 3)

To answer the third research question regarding the degree of far transfer to real-world outcomes, results are heterogeneous (while there is robust evidence for near transfer) and less conclusive than near-transfer outcomes. Across the nine selected studies, only three successfully demonstrated far transfer effects (Studies 2, 3 and 9), showing that benefits extended to skills not directly rehabilitated by the SG, such as academic fluency or complex problem-solving.

Detailed insights from specific studies further clarify these transfer dynamics. For instance, Study 8 suggests that improvements in attention span might result from behavioral training, learning to focus on the tablet while ignoring classroom distractors, rather than an intrinsic rehabilitation of cognitive functions. This highlights a significant methodological limitation: the high risk of bias in some studies (e.g., Study 8) may lead to an overestimation of far-transfer effects that do not reflect stable clinical progress. Similarly, Study 6 noted that while objective tests showed significant near transfer, clinical ratings did not reflect a reduction in ADHD symptoms in daily life. This suggests that while SG may have a motivational impact and improve trained skills, this alone may not be sufficient to reduce ADHD symptoms that continue to affect daily life.

This suggests that without metacognitive bridging or hybrid models that combine SGs with adult-led coaching, as seen in the successful far-transfer of Study 9, digital gains remain encapsulated within the game environment. On the other hand, study 9 provides a successful example of far-transfer to math fluency and the spontaneous adoption of metacognitive strategies suggesting that stimulating key components like WM and Selective Attention within a structured intervention can bridge the gap to academic skills. Moreover, in Study 1, although far transfer effects were observed, it is non-specific, because similar improvements were also recorded in the CG, likely due to placebo effects or the benefits of a structured routine. Conversely, two studies explicitly failed to find significant improvements in far transfer measures (Studies 5 and 6), and for another two studies, this information was either unavailable or not measured. In the case of Study 5, despite a multi-component design targeting various EFs, the lack of far-transfer suggests that in populations with Intellectual Disabilities, cognitive gains may remain strictly encapsulated within the trained domains. In one specific case, far transfer could only be qualitatively evaluated through descriptive parent and teacher reports, which limit finding generalization (Study 7).

This inconsistency in far-transfer requires deeper mechanistic reasoning, as the long-term aim of EFs training is to improve a child's everyday functioning, including academic and social skills. However, these complex competencies are not governed by a single process, but emerge from a dynamic interplay between multiple EFs (89). The generalization gap observed in our review aligns with meta-analytic evidence (90, 92), which confirms that while near-transfer is robust, far-transfer to distal skills like mathematics, literacy, or fluid intelligence is often negligible. A primary explanation for this failure, as highlighted by Kassai et al. (91), is the independence of EFs Components. This hypothesis suggests that WM, Inhibition, and Cognitive Flexibility are independent constructs that mature in parallel rather than being linked by a direct causal relationship. Consequently, improving one specific function (e.g., inhibition) does not necessarily spill over into other functions or complex real-world behaviors.

Our data reinforce this perspective: in several cases, gains were strictly task-specific. For instance, Study 1 showed that visuospatial WM improvements occurred only in the targeted condition, while inhibitory and interference control increased in both Full-Active and Partially-Active group confirming that EFs may operate through distinct functional architectures.

To provide more theoretical depth to the discussion, it is essential to consider the Task Impurity problem and the functional independence of EF components (91). Since most tests do not isolate a single function (e.g., a flexibility task inherently requires inhibitory control), the cognitive load in many SGs may be too fragmented to produce stable, generalizable changes. The evidence synthesized here suggests that SGs are promising tools for improving core cognitive processes, but their ability to foster generalization to real-world outcomes relies on a dynamic interplay between cognitive load, game mechanics, and the pedagogical scaffolding provided by the environment. Another critical factor is the specificity of current SG designs. Many interventions examined in this review focus on a single EF component. However, as suggested by Kassai et al. (91), training a single component is often insufficient to trigger the neural synergy required for practical benefits.

### 4.6 Stability of effect and long-term sustainability

The stability of these gains remains a critical unanswered question: most studies did not include long-term training or follow-up assessments to evaluate the stability of these gains. This evidence aligns with the challenges recognized in the literature regarding the difficulty of demonstrating long-term transfer of skills acquired in the game to real life. The lack of long-term retention is a significant limitation for the clinical impact of SGs in training EFs. The literature highlights how the lack of standardization in reporting can hinder the replicability and generalizability of results, limiting the scientific understanding of SGs.

As a result, while near-transfer effects are well-documented, no definitive conclusions can be drawn regarding the far transfer potential or the long-term clinical utility of these interventions based on the current evidence. The maintenance of effects over time must be assessed through rigorous follow-up. Notably, only one study included follow-up data, which highlighted the maintenance of cognitive gains three months after the end of the training, preventing us from determining if the observed improvements represent a permanent shift in the developmental trajectory or merely a temporary fluctuation.

### 4.7 Methodological considerations and future directions

The present review suggests that, while SGs for attention and EFs training have a solid foundation in defining and pursuing serious objectives, there are significant challenges in their full implementation and, above all, in documenting their characteristics. SGs are generally well-structured to achieve specific EFs learning or rehabilitation goals, with a focus on objective clarity and task feasibility. Critical challenges arise in rigorous content validation, bias mitigation, and, crucially, long-term retention of EFs learning, which remains an ongoing challenge in SGs research (59, 60). The high frequency of not available information about several methodological characteristics of SGs reported in the present review highlights a systemic gap in SGs description. This lack of detailed information about SGs characteristics limits the scientific community's ability to fully understand how serious and gaming features are implemented, how they influence each other, and what their actual impact on EFs is. To advance the field, it is imperative that future research adopt more rigorous reporting standards, providing explicit details on all aspects of SGs design and implementation.

Our analysis adopts a more critical approach toward actual generalizability compared to recent literature, such as the review by Rodríguez-Timaná et al. (73). While they highlight the general potential of SGs as therapeutic tools for neurodiverse populations, our review evaluates clinical efficacy across multiple cognitive domains, while also considering far transfer and treatment adherence as key indicators of success.

Additionally, our methodological approach addresses some gaps found in existing reviews. For instance, Rodríguez-Timaná et al. (73) limited their search to Open Access articles, potentially restricting the completeness of the clinical picture, and included studies without control groups. By contrast, our selection strictly requires a control group and explores a wider range of publications, ensuring a more rigorous assessment of whether observed improvements are truly attributable to the SGs intervention.

### 4.8 Recommendations for practice and future research

To transition from laboratory efficacy to real-world clinical utility, future research should adopt more rigorous methodological standards. It is fundamental that motivation should be measured directly rather than being inferred from adherence rates, integrating validated psychometric tools. These instruments should be administered at multiple points during the rehabilitative process to monitor the sustainability of the gamification effect and prevent a decline in interest over the long term. In parallel, future manuscripts must include detailed technical descriptions of the active ingredients of the game. Authors should explicitly report the types of feedback used, the adaptivity algorithms for difficulty regulation, and game design elements, such as narrative or social interaction components, as these factors directly moderate cognitive load and treatment efficacy. Furthermore, it would be necessary to develop more comprehensive SGs, capable of simultaneously rehabilitating a greater number of attentional skills and EFs. Moreover, to assess whether SGs produce permanent neuroplastic changes rather than simple temporary performance boosts, it is imperative to include follow-up assessments at three or six months after the intervention ends.

Ultimately, the clinical utility of SGs is not merely a product of the game format but stems from a complex interaction between the target population's cognitive architecture, the precision of the rehabilitative design, and the sustained engagement of the user. Provided that the focus remains on therapeutic objectives rather than purely ludic interaction, SGs represent a powerful, inclusive, and intensive supplementary tool for modern neurorehabilitation.

## 5 Limitations

Several limitations must be considered when interpreting these findings. First, it is important to address the sample size and the search strategy employed. Although only nine studies met the eligibility criteria, this relatively small number reflects a fundamental challenge in the current literature on SGss: the widespread lack of rigorous methodological designs. To ensure maximum sensitivity, our search string included three synonyms of SGs,to capture interventions that might not use the serious label despite meeting our PICOS criteria. The limited final sample is therefore a direct consequence of our strict adherence to inclusion and exclusion criteria, specifically the requirement for an untrained control group and objective pre- and post-intervention assessments. These criteria were prioritized to ensure that any observed cognitive improvements could be reliably attributed to the digital intervention, favoring scientific rigor over the quantity of included papers. This selection highlights the urgent need for more standardized clinical protocols in the field.

The high heterogeneity across studies represents a primary challenge for the generalizability of our conclusions. However, this heterogeneity reflects the intrinsic nature of SGss as flexible clinical tools. As highlighted by Chaldogeridis and Tsiatsos (93), future research must overcome the lack of detailed reporting regarding game structure and specific functioning to allow for better replication. Furthermore, factors such as small sample sizes, the absence of active control groups, and the lack of long-term follow-up should be considered when designing future game-based systems to prevent bias. The variability in intervention modalities and dosages (ranging from 4 to 50 h of total training) further complicates the synthesis of results. Following the framework by Green et al. (78), our findings must be interpreted through the lens of effectiveness rather than pure efficacy, acknowledging that real-world compliance, where users may not maintain the exact frequency or precision of the original protocol, significantly impacts clinical outcomes.

A critical methodological limitation is the almost total absence of follow-up data. This prevents us from drawing conclusions about the long-term effectiveness of SGss. Future research should prioritize longitudinal designs to assess whether the cognitive benefits observed immediately after training are maintained over time.

Moreover, the methodological discrepancy between the cognitive domains targeted by the SGs and those actually assessed through pre- and post- test measures. Although many included studies utilized SGs designed to rehabilitate a broad range of functions (e.g., sustained and selective attention, inhibition, flexibility, and WM), they often limited their empirical evaluation to only a subset of these areas. This selective assessment makes it difficult to determine the effectiveness of these tools in achieving a holistic enhancement of the EFs profile, as many potentially rehabilitated functions remain unmeasured.

Beyond these assessment gaps, there is a significant lack of specificity in the technical descriptions of the SGs used; for instance, the exact modality of feedback (visual, auditory, or multimodal) is rarely specified, and it is often unclear whether SGs contained distractors that could impair or facilitate learning. Moreover, many SGs are implemented for individual use; the potential benefits of multiplayer experiences or multisensory stimulation remain largely unexplored.

While gamification elements such as conflict, feedback, and progression are theoretically designed to boost motivation, many studies provide poor descriptions of these mechanics (93). In many cases, motivation is not explicitly evaluated but is taken for granted due to the game format. However, for a SG to be truly effective, it must incorporate well-defined features such as appropriate challenge levels and immediate feedback (93). The fact that elements of motivation are often neither measured nor described represents a critical issue, as it obscures whether the observed clinical improvements are driven by the rehabilitative protocol or by a transient novelty effect.

The effectiveness of SGs depends on a complex interplay of design factors, including interface, feedback quality, playability, and narrative depth (61–63). Subjective factors related to the user's state of mind, such as immersion and personal interest, also play a critical role (64). Furthermore, this review does not cover non-digital formats such as augmented reality or robotics, nor does it evaluate the impact of SGs on emotional regulation or social perception. The results cannot be generalized to other age groups, such as university students or the elderly, who may show different response patterns. Finally, the lack of systematic, objective metrics for motivation in the majority of the sample limits our current understanding of the long-term sustainability of these digital interventions.

Our review focused primarily on 2D digital interfaces; however, evidence from studies comparing 2D and 3D virtual environments (65) suggests that 3D narrative-contextualized SGs can generate lower reaction times, more correct answers, and fewer perseverative responses in attentional abilities and inhibition control. This indicates that more ecological and immersive tools might identify functional cognitive status more accurately than traditional 2D systems. However, any study using 3D environments, meets the inclusion criteria such as the presence of a control group and pre- and post-intervention assessments. Future research should prioritize comparing the efficacy of 2D vs. 3D (Virtual Reality) environments to determine which interface provides superior clinical benefits.

Future research should prioritize RCTs studies, with exhaustive neuropsychological assessment of each attentional and EFs ability and including standardized motivational scales and ecological measures to assess also far transfer effects, as well as long-term follow-ups to verify the persistence of gain.

## 6 Conclusion

This systematic review provides a multi-dimensional map of the current state of SGs for executive rehabilitation. By synthesizing the evidence across the three core research questions, it is possible to draw significant conclusions that integrate cognitive efficacy with engagement dynamics. Regarding Q1 (Cognitive Efficacy), the evidence confirms that SGs are highly effective for rehabilitating inhibitory control and attention across diverse atypical populations, including those with ADHD, intellectual disabilities, and externalizing disorders. Conversely, higher-order functions such as WM and cognitive flexibility appear more resistant to training, suggesting a need for more specialized and adaptive designs to achieve consistent clinical gains. Regarding the long-term sustainability of these interventions, the current evidence remains inconclusive. As only one study in our sample included a follow-up assessment, it is impossible to determine whether the observed improvements in attention and EFs persist after the intervention ends. This highlights a major blind spot in the field: without longitudinal data, we cannot distinguish between transient performance boosts and genuine neuroplastic changes.

In relation to Q2 (Motivation and Engagement), a significant methodological gap has emerged in the current literature. Although gamification is theoretically designed to sustain effort over time, the lack of standardized metrics and detailed technical descriptions prevents a clear understanding of how psychological engagement directly influences cognitive outcomes.

Finally, for Q3 (Near and Far-Transfer), while near-transfer is a robust and consistent finding, far-transfer to academic and behavioral domains remains discontinuous. Successful generalization appears to rely heavily on hybrid intervention models that combine digital training with adult-led coaching, thereby bridging the gap between the game environment and real-life contexts.

SGs represent a promising tool for enhancing attentional functions and EFs in children with typical and atypical development. These tools can be used not only in clinical settings and in telerehabilitation to allow intensive rehabilitation, but also in schools to promote EFs and attentional skills. This presents an opportunity to make training more inclusive (accessible to everyone and every place), intensive and effective. Ensuring accessibility through inclusive design and cultural appropriateness is not merely a technical requirement, but a fundamental ethical obligation to guard (66).More research is needed to develop SGs that train all EFs simultaneously and maintain a high level of motivation in the long term. Special attention should be paid to designing SGs that integrate emotional components and sophisticated gameplay mechanics to maximize the effectiveness of the intervention and ensure lasting results.

## Data Availability

The original contributions presented in the study are included in the article/supplementary material, further inquiries can be directed to the corresponding author.
